# Biomolecular Liquid–Liquid Phase Separation for Biotechnology

**DOI:** 10.3390/biotech12020026

**Published:** 2023-04-01

**Authors:** Sumit Shil, Mitsuki Tsuruta, Keiko Kawauchi, Daisuke Miyoshi

**Affiliations:** Faculty of Frontiers of Innovative Research in Science and Technology (FIRST), Konan University, 7-1-20 Minatojima-minamimachi, Chuo-ku, Kobe 650-0047, Hyogo, Japan

**Keywords:** liquid–liquid phase separation, biomolecules, biotechnology, drug delivery, biocatalysts, drug protection

## Abstract

The liquid–liquid phase separation (LLPS) of biomolecules induces condensed assemblies called liquid droplets or membrane-less organelles. In contrast to organelles with lipid membrane barriers, the liquid droplets induced by LLPS do not have distinct barriers (lipid bilayer). Biomolecular LLPS in cells has attracted considerable attention in broad research fields from cellular biology to soft matter physics. The physical and chemical properties of LLPS exert a variety of functions in living cells: activating and deactivating biomolecules involving enzymes; controlling the localization, condensation, and concentration of biomolecules; the filtration and purification of biomolecules; and sensing environmental factors for fast, adaptive, and reversible responses. The versatility of LLPS plays an essential role in various biological processes, such as controlling the central dogma and the onset mechanism of pathological diseases. Moreover, biomolecular LLPS could be critical for developing new biotechnologies such as the condensation, purification, and activation of a series of biomolecules. In this review article, we introduce some fundamental aspects and recent progress of biomolecular LLPS in living cells and test tubes. Then, we discuss applications of biomolecular LLPS toward biotechnologies.

## 1. Introduction

**Figure 1:**
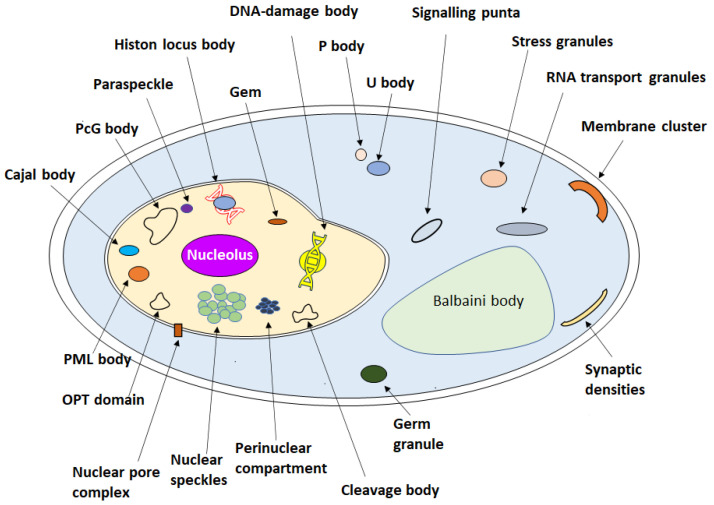
Schematic representation of eukaryotic cell and various biomolecular droplet observed inside the cytoplasm, nucleolus, and cell membranes. Certain droplets are unique to particular cell types. For example, balbiani bodies and germ granules are unique to germ cells, and RNA transport granules and synaptic densities are unique to neuronal cells.

A living cell is made up of different kinds of organelles. Some of these are covered by membranes and some of these are membrane-less [1,2]. Almost one century ago, British scientist J. Haldane (1929) and Russian biochemist A. Oparin (1965) independently proposed the first genesis of life theory. Both theories, incidentally, describe the original concept of prebiotic compartmentalization. According to Oparin, the earliest polymers that resembled proteins and carbohydrates would form into colloidal particles, and these particles would subsequently evolve a rudimentary metabolism resembling fermentation. He postulated that after other nutrients became rare, the colloidal particles would start to develop photosynthesis and absorb the available resources through fermentation. These particles, known as “coacervates”, are organic-rich droplets produced by LLPS [3]. Despite such great proposals for the origin of the prebiotic compartmentalization with the coacervates, the membranelles organelles in living cells have very recently been discovered compared with the membrane organelles [4]. Examples of some membrane-less organelles include the stress granule, Nucleolus, P-body, U-body, Gem granule, Cajal body, and Histone locus body (Figure 1) [5]. These are formed through the LLPS of biomolecules such as proteins and nucleic acids [6].

LLPS can be useful in controlling (activation and suppression) many important cellular reactions because it can significantly increase the local concentration of the participating molecule in the cellular reaction [7,8]. For example, LLPS is important for a variety of biological processes, including ribosomal biogenesis [9], transcription [10], cell signaling [11], stress response [12], cytoskeletal regulation [13], cell polarization [14], cytoplasmic branching [15], nucleolus formation [16], miRISC assembly [17], innate immune signaling [18], stress granule assembly [19], and autophagy [20]. Furthermore, LLPS has a significant role in various diseases such as cancer [21], Alzheimer’s disease [22], Parkinson’s disease [22], Huntington’s disease, and amyotrophic lateral sclerosis [23,24,25]. Importantly, recent studies have demonstrated that biomolecular LLPS can also be imposed in various applied fields of biotechnology, synthetic biology, food technology, environmental engineering, etc. In this review, we discuss the recent trends in biomolecular LLPS research and its application.

## 2. Principles of Biomolecular LLPS

**Figure 2:**
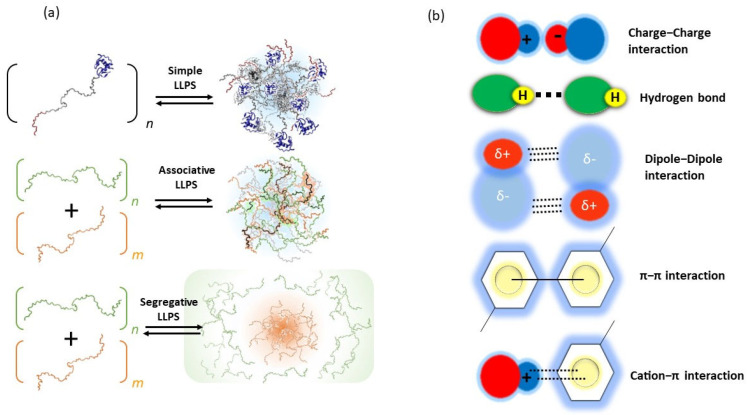
(**a**) Schematic representation of three different types of LLPS and the formation droplet. (**b**) Schematic representation of different kinds of interaction involved for undergoing LLPS.

**Figure 3:**
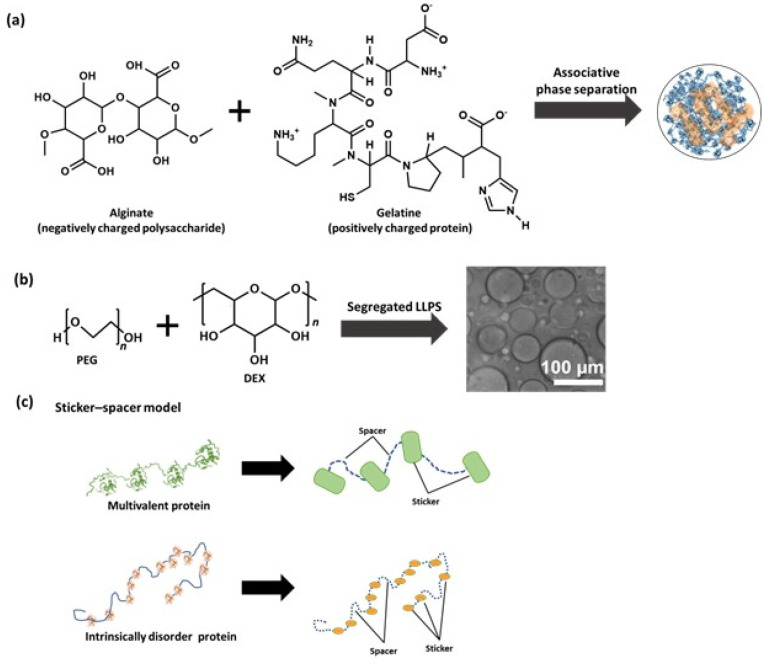
(**a**) Schematic representation of associative phase separation where a negatively charged alginate reacts with positively charged gelatine and produces droplets through associative phase separation. (**b**) Schematic representation of segregative phase separation induced by PEG (Poly-ethylene glycol) with DEX (Dextrin) to generate an artificial droplet. (**c**) Schematic representation of multivalent protein and intrinsically disordered protein as the sticker.

A multi-component system, such as cellular cytoplasm, can exist as a homogenous and well-mixed mixture or a soup of separate phases, depending on the interactions of the constituent molecules, including the solvent that is water in the case of living cells [3]. There are three types of LLPS that can commonly be distinguished (Figure 2a) [26]. In a simple LLPS, a single molecular attractive interaction proceeds that generate coactivates depending on molecular environmental factors, such as temperature, pH, and salt concentration. Many proteins with disordered regions have been found to undergo simple LLPS, driven by a combination of electrostatic interaction and other weak interactions such as hydrogen bonding, π–π stacking, cation–π, and dipole–dipole interactions (Figure 2b) [26]. In associative LLPS, two soluble molecules end up in the same phase, due to attractive interactions between them. This condensed phase is called a droplet; it is enriched in both solutes, but still contains a significant amount of solvent. A classic example of associative LLPS is that of two oppositely charged polymers, such as alginate (a negatively charged polysaccharide) and gelatin (a positively charged protein) (Figure 3a) [26]. In segregative LLPS, two soluble molecules (e.g., peptides, polymers, and nucleotides) do not mix despite a favorable mixing entropy due to repulsive interactions between them. As a result, they end up in two separate phases, each enriched in one of the solutes. A classic example of segregative LLPS is that of poly(ethylene glycol) and dextran (Figure 3b) [26].

Proteins are the primary cause of biomolecular LLPS. Notably, several multi-domain protein systems display LLPS behavior [27,28,29]. As shown in Figure 3c, the so-called stickers-and-spacers approach can be used to conceptually examine multi-domain proteins and intrinsically disordered proteins (IDPs) [29,30]. IDPs have less defined three-dimensional structures in physiological conditions [31]. It was discovered that significant amounts of intrinsically disordered regions (IDRs) can be found in many LLPS systems discovered in living cells [32]. The stickers-and-spacers model divides the target protein into two regions: molecular fragments responsible for chain–chain interactions (stickers), and the remainder of the molecule, which is not involved in the interaction (spacer). Although spacers are suggested to modify chain characteristics, they have a smaller impact on chain–chain interactions than stickers do. Multi-domain proteins can easily undergo LLPS because interacting domains serve as stickers and disordered linkers serve as spacers. Recently, scientists have discovered that divalent ions have some important role in the formation of biomolecular droplets [33]. Zinc ions, which greatly increase the tendency for tau to undergo LLPS by reducing the critical concentration of protein, were found to influence the LLPS of tau protein [34]. Both directly and through interactions with other proteins, divalent cations can influence phase transitions. Recent studies have shown that the EF-hand domain protein (EFhd2) directly affects tau’s liquid phase behavior to form solid-like structures in vitro. This modification is caused by calcium ions [35]. 

## 3. Biomolecular Droplets and Their Functions

The number of biomolecular droplets produced via LLPS is rapidly growing, and their biological functions have been identified [36,37]. In this section, we present biomolecular droplets which occur naturally via LLPS, as well as artificial biomolecules which undergo LLPS to form droplets.

### 3.1. Droplets in Cytoplasm

Biomolecular droplets which are present in the cytoplasm have been identified. Stress granules are a typical example and are the most extensively studied in the cytoplasmic droplets. Stress granules are membrane-less organelles, ranging in size from 0.1 to 2 μm [38]. The essential components for stress granule formation are T-cell-restricted intracellular antigen-1 (TIA-1) and Ras-GTPase-activating protein SH3-domain-binding protein 1 (G3BP1) and RNAs. The primary function of stress granules is to promote cell survival by condensing translationally stalled mRNAs, ribosomal components, translation initiation factors, and RNA-binding proteins (RBPs). On the other hand, certain transcripts such as heat shock protein 70 are excluded from stress granules which are selectively translated under the stress conditions [39]. Therefore, stress granules can control protein expression (translation) through the inclusion and exclusion of certain mRNAs in response to unfavorable conditions for cells. Stress granules are formed under acute stress conditions such as hypoxia, oxidative stress, osmotic stress, and temperature change [40]. The timescale of the disassembly of stress granules varies depending on the stress factors. For example, cold-shock-induced stress granules disassemble within minutes after returning to normal temperature [41]. On the other hand, recovery after arsenate stress, H_2_O_2_ treatment, osmotic stress, or heat shock occurs between 60 and 120 min [42]. In addition to the recovery time varies, this range of time is much shorter than the gene expression response. Therefore, LLPS including the assembly of stress granules is critical for promptly controlling cellular functions to protect cells from death under adverse conditions. Moreover, stress granules under stress conditions alter nuclear events, providing a linkage between the nuclear and the cytoplasmic processes [41]. Stress granules also respond to diseases such as viral infections and cancer [43]. Stress granules are further recognized as potential precursors of pathological aggregates in neurodegenerative diseases [44]. The position, function, chemical composition, and detection technique of cytoplasmic droplets are briefly listed in Table 1 [45,46,47,48,49,50,51,52,53,54,55,56,57,58,59,60,61,62,63].

### 3.2. Droplets in Nucleus

The interior of a cell nucleus is a complex environment: a crowded mixture of biomolecules, including very long DNA strands in the form of chromatin with histone proteins, mRNAs that are newly transcribed, other RNAs for controlling gene expressions, and proteins for transcription and other processes. A wide variety of droplets are required to proceed biologically critical reactions under the complex environment. 

One of the most well-known cellular droplets is the nucleolus, which, in the 1830s, was the first membrane-less component to be identified [5]. The number (usually 2–5 per cell) and size of the nucleoli depend on the cell type, cell cycle phase, and metabolic conditions. The nucleolus provides a site for the transcription of ribosomal RNA from ribosomal DNA and ribosome assembly for ribosome biogenesis. The nucleolus also serves other processes, such as maintaining cell homeostasis [64]. Recently, new roles of the nucleoli have attracted attention: as stress granules, the nucleoli act as sensors and regulators for cellular stresses such as RNA polymerase I inhibitors, prevalent cytotoxic agents, viral proteins, UV radiation, heat shock, and DNA damage, apoptosis, and senescence [65]. 

A nucleolus contains several functional modules, each constituting three sub-compartments or layers. From the inner to the periphery, the three layers are the fibrillar center, the dense fibrillar component, and the granular component, responsible for different steps of ribosomal biogenesis. The nucleolus is composed of hundreds of copies of ribosomal genes, newly synthesized ribosomal RNA (rRNA), ribosomal proteins, and ribonucleoproteins. Other droplets found in the nucleus are listed in Table 2 [8,66,67,68,69,70,71,72,73,74,75,76,77,78,79,80,81,82,83,84,85,86,87,88,89,90,91,92]. 

### 3.3. Droplets in Membranes

Although they do not occur via LLPS, in cell membranes, biomolecular droplets are also produced, such as membrane clusters, as listed in Table 3 [13,28,93,94,95,96,97,98,99,100,101,102,103,104,105]. A membrane cluster is a lipid droplet consisting of triacylglycerols, phospholipids, sphingolipids, cholesterol, and proteins [106]. These clusters play important roles in various cellular processes, including signaling, and the transport of cell take and cell release material such as lipids, amino acids, ions water, and hormones, amines, and peptides [93]. The membrane cluster has a significant role not only in the uptake of lipids, but also in the distribution and storage of lipids. A representative example of a membrane cluster is the photosystem II (PSII) complex, which is involved in the light-dependent reactions of photosynthesis in plants, algae, and some bacteria [107]. PSII is a large and complex protein complex that contains over 20 different subunits, most of which are membrane-bound. It consists of a core antenna complex that captures light energy, a reaction center that uses this energy to split water into oxygen, and electron carriers that transfer the electrons to other components of the photosynthetic system [108]. The formation of PSII clusters is essential for their proper functioning. PSII clusters help to organize the various components and to create a favorable environment for the transport of electrons [109].

### 3.4. Enzymes and Transcription Factors Undergoing LLPS

Recent studies suggest that some enzymes show different activity inside droplets. For example, Saini et al. recently discovered that macromolecular crowding induces LLPS, which leads to an increase in the intrinsic catalytic efficiencies of horseradish peroxidase (HRP) and glucose oxidase (GOx) [110]. Transcription factors (TFs) and RNAs also induce the formation of transcriptional condensates via LLPS, which contain clusters of multiple enhancers (super-enhancers) [111]. This phenomenon is supported by the dynamic interaction of TFs with RNA polymerase II (Pol II) clusters [112]. To form transcriptional condensates, TFs bind to various cis-regulatory DNA elements (e.g., promoters and enhancers) and stimulate the transcription of active genes in proximity, facilitating the precise control of gene expression [113]. Other examples of enzymes and transcription factors which undergo LLPS are listed in Table 4 [110,114,115,116,117,118,119,120,121,122,123,124,125,126,127,128,129,130,131,132,133,134,135,136,137,138]. 

### 3.5. Droplets Discovered in Various Biological Processes

The list of biomolecular condensates is increasing rapidly. For example, rubisco (pyrenoids) plays a crucial role in photosynthesis acceleration and in carbon fixation [139,140,141]. Another interesting droplet recently found is the Wnt droplet [142]. The Wnt droplet consists of proteins such as kinase that regulate β-catenin stability. Wnt droplets play a vital role in stem cell differentiation. These findings demonstrate that LLPS is pivotal and versatile not only in controlling the central dogma, but also in various biological processes. Therefore, it is considerable that LLPS is one of the fundamental characteristics of biomolecules. The location, name, component, biological role, and observation procedure of droplets discovered in various biological processes are listed in Table 5 [139,140,141,142,143,144,145,146]. 

### 3.6. Artificial Droplet System

Artificial and model droplet systems are gaining popularity. Artificial and model droplet systems have various uses because of their controllable size, concentration inside the droplet, and the component of the droplet. Researchers are focusing on the development of new artificial droplets as well as artificial systems. Artificial cells are simplified models of living cells for investigations of the molecular basis of life. Artificial cells are generally constructed using a water-in-oil (W/O) microdroplet. Water in an oil microdroplet is a micrometer-sized water droplet dispersed in an immiscible oil phase [147]. Another artificial droplet system that gains immense popularity is the droplet reactor system. Droplet reactor systems have considerable biochemical applications such as single-cell analysis, kinetic study, and controlled drug release [148]. Another interesting example of an artificial droplet system is DNA nanostructures. DNA nanostructures were employed by Sato and Takinoue to induce LLPS [149]. The DNA nanostructures localize at the oil–water interface when they are added to the oil–water system. Different two-dimensional phase separation patterns could be induced depending on the DNA sequences. Hydrogels were created as a result of the DNA nanostructures’ phase separation [149]. 

Recently, a novel class of short peptide derivatives that undergo LLPS has been created [150]. The peptide is made up of phenylalanine dipeptides joined by hydrophilic spacers (cystamine moiety). Disulfide bonds formed among spacers enable redox-chemistry-based dynamic regulation of the assembly. Additionally, researchers might functionalize the coacervates to act as a catalyst in the aldol and hydrazone production reaction [150]. Other examples of model droplet systems are given in Table 6 [151,152,153,154,155].

## 4. Biomolecular LLPS towards Biotechnology

**Figure 4:**
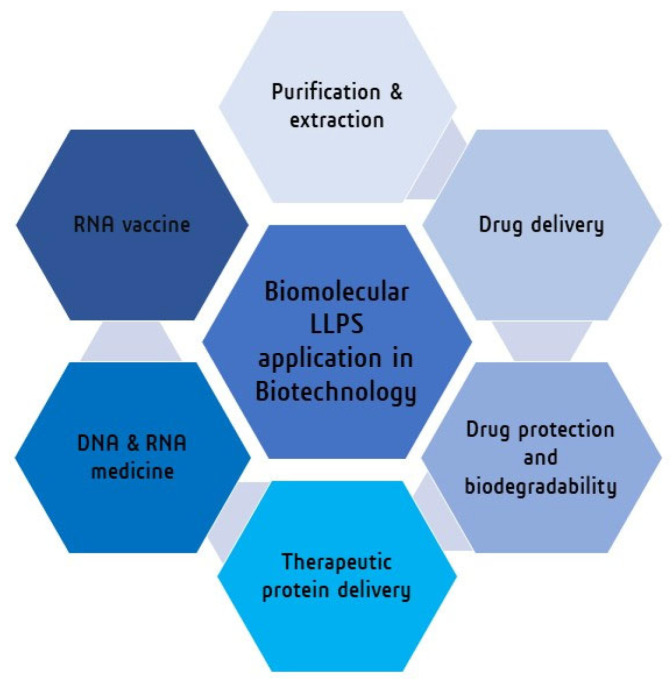
Schematic representation of the application of biomolecular LLPS in various field of biotechnology.

Due to its special characteristics, such as the differential partitioning and compartmentalization of biomolecules, biodegradability, and biocompatibility of biomaterials, LLPS has been used in biotechnological and biomedical processes [156,157,158,159]. Some examples of biomolecular LLPS applications in biotechnology and the biomedical process are given in Table 7 and schematically shown in Figure 4. The chemistry of the three different LLPS systems, simple phase separation, segregation, and associative phase separation, varies from one to the other. In the case of simple phase separation, separation happens based on the density and other physical properties. For segregation phase separation, separation happens based on the size, shape, or charge of the components. In the case of associative phase separation, separation happens based on specific molecular interactions. The affinities for different chemical compounds to participate in phase-separated droplets are strongly influenced by the chemical component and the nature of both the LLPS system’s constituent parts and the chemicals. To account for this, each molecule and LLPS system pair has a “partition coefficient”, which is a parameter of how likely a molecule is to segregate into a droplet in comparison to the surrounding solution. The partition coefficient is more precisely defined as the ratio of the concentration of the molecule outside the droplet to the molecule inside the droplet in an equilibrium state. The partition coefficients of numerous compounds in phase-separated systems have been extensively studied [160,161,162,163,164,165,166]. Bioprocess researchers and engineers have made use of the differential partition coefficients of molecules in diverse LLPS systems as a method for selective biomolecule extraction and purification, which are processes frequently used in commercial biomolecule productions [167]. Recent discoveries demonstrated that biomacromolecules, such as nucleic acids, can be purified based on their phase separation behavior. It was discovered that an ATPS system (PEG-phosphate) in the purification of plasmid DNA from pure E. coli lysate had a yield of 80–85% of plasmid DNA [168]. Using a similar system, it was possible to separate plasmid DNA from RNA (from a mixture that solely contained plasmid DNA and RNA) with yields of 89% (plasmid DNA) and 70% (RNA), depending on pH [169]. 

It was demonstrated that LLPS is not only very useful for biomolecule separation and purification systems, but also for drug delivery systems. Small-molecule drugs, many of which are not easily soluble in aqueous solution, have been compartmentalized using phase-separated droplets [3,170,171]. An additional good reason to use LLPS for drug delivery is that it offers drug protection from biodegradations. Sodium-alginate beads combined with a coacervate system are used in the encapsulation of small-molecule drugs to protect the drug against low pH levels generated by stomach fluids, allowing the drug to be released further downstream in the digestive tract [162]. Such an application of LLPS could be used to prevent drugs from degrading before they reach their destination (target cells) and could improve the efficacy of the drug formulation without altering the chemical structure of the molecule [172]. Moreover, coacervates can provide encapsulated proteins with protection from temperature, pH, and even denaturants such as urea [173]. For example, Nojima et al. produced phase-separated protein condensate with a high concentration of over 600 distinct proteins while maintaining their natural structure using ionic surfactants [174,175]. Due to the protection ability of LLPS from biodegradation, drug delivery systems based on the LLPS of artificial polymers have been reported. Polyester microspheres are an example of such an artificial polymer system which can be charged with a variety of small-molecule medicines [176,177]. Other examples of artificial polymer systems which demonstrate LLPS are polylactate, polyglycolate, poly(lactate/glycolate) co-polymers, and other aliphatic polyesters [178,179,180]. These polymers are composed of simple aliphatic residues, and a straightforward hydrolysis mechanism allows them to break down into their monomer species (lactic acid and/or glycolic acid). The biocompatibility and biosafety of such polymers are suggested by the fact that lactic acid can be safely degraded by a number of natural processes, including neuron metabolism, glucogenesis in the liver, oxidation to pyruvate in muscle cells, or excretion through urine or breath as carbon dioxide [181,182]. Current research is looking at new ways to control the rate of degradation, such as the addition of other polymers [183], the development of novel polyester materials, such as foams [184], or the engineering of polyesters that may selectively degrade in response to specific external stimuli [185]. On the same note, currently, we have seen a substantial increase in therapeutic protein therapies with LLPS. One such demonstration even delivered functional myoglobin to human stem cells using amylose-based coacervates [186]. When more effective protein therapies are developed, the usage of phase-separated protein complexes may become even more prevalent [187]. Some recent research showed that large biomacromolecules, such as antibodies, can be delivered into the cytosol through coacervation using a peptide. T. Iwata et al. discovered that a mixture of Alexa488-IgG and FcB(L17E)3 formed liquid droplets; the hydrophobicity and basicity of the Alexa488-IgG and FcB(L17E)3 segment are important for liquid droplet formation [188]. These findings open a new horizon for small drugs and protein delivery inside cells. In recent years, scientists have utilizing the LLPS system for personalized genetic medicine treatments, such as DNA or RNA medicine, including miRNA and siRNA therapy [189,190,191,192,193]. Currently, mRNA vaccines against SARS-CoV-2 use lipid nanoparticles [194,195], and early LLPS systems have demonstrated the capacity to concentrate or shelter nucleic acids [119]. A more recent study has started to concentrate on the stabilization of encapsulated viruses (as vaccine vectors) in coacervates [196]. Future studies might be interestingly directed toward using LLPS systems as genetic carriers in RNA or DNA vaccines. Despite having all these applications, biomolecular LLPS also have other application in diverse field of biotechnology. LLPS plays an important role in material science. Recently, scientists have discovered that, by applying LLPS systems we can form new materials such as microgels [197]. Biomolecular LLPS systems have potential applications in environmental science. In a recent study, Zhou et al. efficiently removed organic pollutant from water using poly-lipoic ester base coactivates [198]. Another example of the application of LLPS systems in environmental science is the removal of phosphate from wastewater; environmental scientists have used microorganisms to extract phosphorus from wastewater by taking advantage of their proclivity to incorporate exogenous phosphates into internal phase-separated organelles [199]. In addition to these applications, biomolecular LLPS systems have applications in the food industry. One of the most common uses of LLPS is to create desirable textures in food products. LLPS causes the production of W/W emulsions containing biopolymers dispersed disproportionately between separated liquid phases [200]. Another use of LLPS systems in the food industry is the encapsulation of ingredients [201]. LLPS based on carbohydrates or proteins is often utilized to encapsulate active substances within the dispersion phase. In food items, encapsulation serves several functions, including hiding undesirable odors, stabilizing reactive substances, and managing the release of active compounds [202].

**Table 7:** Biomolecular LLPS systems and their application in biotechnology.

Biomolecular LLPS System	Application in Biotechnology	Ref.
ATPS system (PEG-phosphate)	Purification and extraction of biomolecules (DNA, RNA)	[168,169]
Sodium-alginate beads combined coacervate system	Drug protection	[162,173,174,175,176]
Polyester microspheres and artificial polymer system	Drug delivery	[177,178,179,180,181,182,183,184,185,186]
Amylose-based coacervates	Protein delivery	[187,188,189]
Lipid nanoparticles	DNA, RNA vaccines	[190,191,192,193,194,195,196,197]
Poly-lipoic ester base coactivates	Organic pollutant remover	[199]
LLPS system from the gel–sol transition of protein (gelatin solution) in a macromolecular crowding agent (PEG solution).	New material synthesis (protein microgel)	[198]
Carbohydrates and proteins-based LLPS system	Encapsulation of active substances (food industry)	[201,202]

## 5. Conclusions and Perspectives

The importance of biomolecular LLPS and its roles in diverse biological processes is evident, as well as its involvement in a series of diseases. This review has provided an overview of biomolecular LLPS systems across various applied fields of biology, including biotechnology and synthetic biology. The unique properties of LLPS systems, such as their ability to concentrate biomolecules, organize biochemical reactions, and generate membrane-less organelles, make them ideal candidates for a wide range of applications, including drug delivery, enzyme immobilization, and biocatalysis. However, there are still many challenges to be addressed for practical applications of LLPS in biotechnology, such as controlling the size and stability of the resulting droplets, optimizing the conditions for LLPS, and scaling up production. More rational design and controlling procedures of LLPS are highly required. Another area of future research is the integration of LLPS systems into more complex biotechnological processes, such as bioreactors or biosensors, because the selective enrichment of molecules inside droplets could be useful as a pretreatment of reactors and sensors. Additionally, the development of high-throughput screening methods based on biomolecular LLPS could enable the discovery of new functional molecules involving small- to large-sized drugs targeting biomolecular droplets. Such drugs controlling the LLPS of biomolecules will be a new modality for broad spectrums of diseases. Overall, the potential of biomolecular LLPS for biotechnology is vast, and continued research in this field is likely to lead to significant advances in the coming years.

## Figures and Tables

**Table 1:** Components, roles, and observation procedures of droplets in cytoplasm.

Droplet	Main Component	Role	Observation	Ref.
Stress granule	Proteins and RNAs	Translational regulation mRNA storage	CM ^(1)^, smFISH ^(2)^	[42,43]
Centrosome	Pericentriolar material	Formation of mitotic spindles during mitosis	CM ^(1)^	[44,45]
U body	Uridine-rich small nuclear ribonucleoproteins	Storage and assembly of snRNPs	CM ^(1)^	[46,47]
G body	Lipid and protein	Controlling the rate of glycolysis	CM ^(1)^	[48,49]
P body	Translationally repressed mRNAs and proteins related to mRNA decay	mRNA decay and silencing	CM ^(1)^	[50,51]
Balbiani body (germ cells)	Endoplasmic reticulum/Golgi-like vesicles, mitochondria, and specific RNAs transporter.	Storage and facilitating the organization of the oocyte into a polarized cell	CM ^(1)^, EM ^(3)^	[52,53,54,55]
Germ granules (germ cells)	Proteins and RNAs	Storage of proteins and RNAs that are required for germ cell development	EM ^(3)^	[56,57,58]
RNA transport granule (neuronal cell)	mRNAs and proteins	Storage and transport of mRNAs	CM ^(1)^	[59,60]

^(1)^ CM, confocal microscopy; ^(2)^ smFISH, single molecular fluorescence in situ hybridization; ^(3)^ EM, electron microscopy.

**Table 2:** Components, roles, and observation procedures of droplets in the nucleus.

Droplet	Main Component	Role	Observation	Ref.
Nucleolus	Proteins and canonical nucleic acids, non-coding RNA	Ribosome biogenesis	FM ^1^	[8,63,64]
Histone locus body	NPAT ^10^, SLBP ^11^, the U7 spliceosomal snRNP-specific components, such as Sm proteins, LSm10 and LSm11, and the U7 spliceosomal snRNA, FLASH ^12^	Histone mRNA biogenesis	BFM ^2^	[65,66]
Heterochromatin	HP1 ^13^, nucleosomal DNA	Promote the formation of heterochromatin	CM ^3^	[67,68,69,70,71,72]
Nuclear pore central transport channel and nuclear pore complex	Nups ^14^, FG ^15^	Chromosomal translocations, change in protein expression levels. Fuse with oncoproteins, nuclear import/export	HS-AFM ^4^, FM ^1^	[73,74,75,76]
Nuclear speckles	RNAs and proteins	mRNA splicing	CM ^3^	[77,78]
DNA damage foci	Rad52 DNA repair proteins	DNA damage repair	Live-cell CM ^5^	[79,80]
Gem	SMN complex, ZPR1, GEMIN2–8 ^16^.	Storage aid histone, mRNA processing	CM ^3^	[81,82,83]
PcG body		Transcriptional repression	IM ^6^, EM ^7^	[84,85]
Paraspeckle	NONO ^17^, PSP1 ^17^, PSP2 ^17^, SFPQ ^18^, CFIm68 ^19^, CFIm, hnRNPs ^20^ NEAT1 ^21^	RNA processing	CM ^3^	[86,87,88]
OPT domain	The RNA polymerases and the general transcription factors	Transcriptional regulation	FM ^1^, EM ^7^	[89,90,91]
Cajal body	Coilin, CB-specific RNAs	Assembly and/or modification of splicing machinery	BFM ^2^	[66,92,93]
Perinuclear compartment	RNA-binding proteins and pol III RNA	Associated with malignancy	EM ^7^, IM ^6^	[92]
Cleavage body	snRNPs ^22^, p80-coilin protein, RNA polymerases, transcriptional factors, nucleolar constituents	mRNA processing	IL ^8^	[94]
Nuclear bodies (NBs)	Protein and non-protein components, heat shock transcription factors, HSF1 ^23^ and HSF2 ^24^, SAF-B ^25^, Sam68 ^26^, SRSF1 ^27^, SRSF7 ^27^ and SRSF9 ^27^. RNA Pol II.	Regulation of genome function	IM ^6^, SRM ^9^	[66,95]
PML body	DAXX, SUMO ^28^	Transcriptional regulation; apoptosis signaling; antiviral defense	EM ^7^	[82,96]

^1^ FM, fluorescence microscopy; ^2^ BFM: bright-field microscopy; ^3^ CM, confocal microscopy; ^4^ HS-AFM, high-speed atomic force microscopy; ^5^ live-cell CM; ^6^ IM, immunofluorescence microscopy; ^7^ EM, electron microscopy; ^8^ IL, immunofluorescence labelling; ^9^ SRM, super-resolution microscopy; ^10^ NPAT, factors required for processing histone pre-mRNAs, nuclear protein, ataxia–telangiectasia locus; ^11^ SLBP, stem-loop binding protein; ^12^ FLASH, FLICE-associated huge protein; ^13^ HP1, heterochromatin protein; ^14^ Nups, nucleoporins; ^15^ FG, phenylalanine–glycine; ^16^ GEMIN2–8, gem-associated proteins 2–8; ^17^ NONO, PSP1, PSP2, paraspeckle component proteins; ^18^ SFPQ, splicing factor proline/glutamine-rich; ^19^ CFIm68, mammalian cleavage factor I 68; ^20^ hnRNPs, Heterogeneous nuclear ribonucleoproteins; ^21^ NEAT1, long ncRNA nuclear-enriched abundant transcript 1; ^22^ snRNPs, small nuclear ribonucleoproteins; ^23^ HSF1, heat shock factor 1; ^24^ HSF2, heat shock factor 2; ^25^ SAF-B, scaffold attachment factor B; ^26^ Sam68: Src-associated mitosis 68 kDa protein; ^27^ SRSF1, SRSF7, SRSF9, SRSF family members; ^28^ SUMO, a potent repressor of transcription and modulator of apoptosis, ubiquitin-like protein.

**Table 3:** Components, roles, and observation procedures of droplets in membranes.

Droplet	Main Component	Role	Observation	Ref.
Membrane cluster	Triacylglycerols, phospholipid, protein	Lipid uptake, distribution, storage, and use in the cell.	-	[98]
Synaptic densities	Actin’s cytoskeleton, kinases, phosphatases, and regulators, GTPases, subunits of AMPA and NMDA receptors, Catenin, N-Cadherin	Neurotransmission	AEM ^1^	[99,100]
Focal adhesions	p130Cas (‘Cas’) and FAK ^7^	Cell adhesion/migration	SDCM ^2^	[101,102]
Nephrin clusters	Cytoplasmic adaptor protein Nck, the nephrin–Nck–N-WASp complex	Glomerular filtration barrier	SRSIM ^5^	[13,28,103]
TCR clusters	LAT ^8^	Immune synapse	TIRF ^3^	[104,105,106]
Podosomes	F-actin and its regulatory molecules, structural proteins	Cell adhesion/migration	PCM ^4^	[107,108]
Actin patches	Actin-associated proteins, upstream signaling molecules	Endocytosis	EM ^6^	[109,110]

^1^ AEM, advanced electron microscopy; ^2^ SDCM, spinning disk confocal microscopy; ^3^ TIRF, total internal reflection fluorescence microscopy; ^4^ PCM, phase-contrast microscopy; ^5^ SRSIM, super-resolution structured illumination microscopy; ^6^ EM, electron microscopy; ^7^ FAK, focal adhesion kinase; ^8^ LAT, linker protein for activation of T cells.

**Table 4:** Components, roles, and observation procedures of droplet enzymes.

Enzyme	Role	Observation	Ref.
Horseradish peroxidase (HRP)	Catalyst (horseradish peroxidase (HRP)	CM ^1^	[115]
Glucose oxidase (GOx)	Catalyst (oxidation of β-d-glucose to d-glucono-δ-lactone)	CM ^1^	[119]
Hexokinase	Catalyst, catalyzing the phosphorylation of keto- and aldohexoses	OM ^2^	[120]
Lipase	Fat breakdown	CM ^1^	[121,122]
Hammerhead ribozyme	Cleavage and ligation of RNA molecule	FRET ^6^, CD ^4^, CM ^1^	[123,124]
Pfk2, Eno1, Eno2, Fba1	Glycolysis	FM ^3^	[125]
GIT1	GTPase activator	FRAP ^5^, CM ^1^	[126]
HSF1	Transcription factor	FM ^3^	[127,128]
NELFE	Transcriptional regulation	FM ^3^	[129]
p53	Transcription factor	FM ^3^	[130,131,132,133,134,135]
PLK4	Serine/threonine-protein kinase	CM ^1^	[136,137]
SOX-2	Transcription factor	FM ^3^	[138]
TFE3	Transcription factor	FM ^3^	[139]
TFEB	Transcription factor	FRAP ^5^	[140]
USP42	Deubiquitinating enzyme	FM ^3^	[141]
YAP	Transcription factor	FM ^3^	[142,143]

^1^ CM, confocal microscopy; ^2^ OM, optical microscopy; ^3^ FM, fluorescence microscopy; ^4^ CD, circular dichroism; ^5^ FRAP, fluorescence recovery after photobleaching; ^6^ FRET, fluorescence resonance energy transfer microscopy.

**Table 5:** Location, components, roles, and observation procedures of droplets discovered in various biological processes.

Droplet	Location	Role	Main Component	Observation	Ref.
Pyrenoids (Rubisco), carboxysomes	Chloroplast	Photosynthesis, metabolism (Carbon fixation)	Carboxysomal linker proteins CsoS2 and CcmM, Rubisco large subunit	Microscopy and sedimentation assay	[144,145,146]
Wnt droplet	Cell cytoplasm	Stem cell differentiation, controlling Wnt pathway	Scaffold proteins and kinases that regulate β-catenin stability	CRISPR-engineered fluorescent tags, optogenetic tools	[147]
YTHDC droplet (nuclear bodies)	Nucleus	AML cell survival, differentiation state, leukemogenesis	YTHDC1 protein, m6 A-containing RNA	IF ^1^, SEM ^2^.	[148]
LDAM ^3^	Hippocampus	Promotion of pathogenesis, neuroinflammation	Lipid	CARS ^4^	[149]
Lipid droplets	Cell cytoplasm (Stem cell)	Skeletal muscle satellite cell fate determination	Lipid	TEM ^5^	[150]
Plant lipid droplets: LD-Erm LD-Peroxisomes	Plant cell	Unknown	Triacylglycerols (TAGs), sterol esters (SEs)	FM ^6^, CM ^7^	[151]

^1^ IF, immunofluorescent imaging; ^2^ SEM, scanning electron microscopy; ^3^ LDAM, hippocampus lipid droplet accumulating microglia; ^4^ CARS, coherent anti-Stokes Raman scattering microscopy; ^5^ TEM, transmission electron microscopy; ^6^ FM, fluorescence microscope; ^7^ CM, confocal microscopy.

**Table 6:** Components, application, and observation procedures of artificial droplets.

Droplet	Component	Observation	Application	Ref.
Adiposomes (artificial lipid droplets (ALDs))	Phospholipids and neutral lipids such as TAG	LM ^1^, EM ^2^	Potential usage in drug delivery.	[156]
Cell-sized aqueous/aqueous microdroplets (CAMDs)	PEG ^3^ and DEX ^4^, actin	FM ^5^	Provide cell-like crowded microenvironments	[157]
Microfluidic platform in a defined pattern	Hexadecane/squalene with dissolved lipids		Broad range of applications in the field of artificial cells, bioreactors, and pharmacological studies.	[158]
Lipase-stabilized tributyrin microcompartment and amylose-polymer-stabilized 2-ethyl-1-hexanol microcompartment	Amy-PNIPAAm ^6^, BSA-PNIPAAm ^7^, Lipase	OM ^8^	Synthetic biology, bottom-up reaction	[159]
G-quadruplex-forming oligonucleotides and R-rich oligopeptides	FMR1 RNA, C9orf72 RNA, peptide derived from FMRP	CM ^9^	Droplet redissolution in a sequence-specific manner	[160]

^1^ LM, light microscopy; ^2^ EM, electron microscopy; ^3^ PEG, poly (ethylene glycol); ^4^ DEX, dextran; ^5^ FM, fluorescence microscopy; ^6^ Amy-PNIPAAm, Poly(N-isopropylacrylamide; ^7^ BSA-PNIPAAm, bovine serum albumin-Poly(N-isopropylacrylamide; ^8^ OM, optical microscopy; ^9^ CM, confocal microscopy.

## Data Availability

Not applicable.
